# The Role of TIM-1 and CD300a in Zika Virus Infection Investigated with Cell-Based Electrical Impedance

**DOI:** 10.3390/bios14080362

**Published:** 2024-07-25

**Authors:** Merel Oeyen, Clément J. F. Heymann, Maarten Jacquemyn, Dirk Daelemans, Dominique Schols

**Affiliations:** Laboratory of Virology and Chemotherapy, Department of Microbiology, Immunology and Transplantation, Rega Institute for Medical Research, KU Leuven, 3000 Leuven, Belgium; clement.heymann@kuleuven.be (C.J.F.H.); maarten.jacquemyn@kuleuven.be (M.J.); dirk.daelemans@kuleuven.be (D.D.); dominique.schols@kuleuven.be (D.S.)

**Keywords:** zika virus, impedance, TIM-1, CD300a, phosphatidylserine receptor

## Abstract

Orthoflaviviruses cause a major threat to global public health, and no antiviral treatment is available yet. Zika virus (ZIKV) entry, together with many other viruses, is known to be enhanced by phosphatidylserine (PS) receptors such as T-cell immunoglobulin mucin domain protein 1 (TIM-1). In this study, we demonstrate for the first time, using cell-based electrical impedance (CEI) biosensing, that ZIKV entry is also enhanced by expression of CD300a, another PS receptor. Furthermore, inhibiting CD300a in immature monocyte-derived dendritic cells partially but significantly inhibits ZIKV replication. As we have previously demonstrated that CEI is a useful tool to study Orthoflavivirus infection in real time, we now use this technology to determine how these PS receptors influence the kinetics of in vitro ZIKV infection. Results show that ZIKV entry is highly sensitive to minor changes in TIM-1 expression, both after overexpression of TIM-1 in infection-resistant HEK293T cells, as well as after partial knockout of TIM-1 in susceptible A549 cells. These results are confirmed by quantification of viral copy number and viral infectivity, demonstrating that CEI is highly suited to study and compare virus-host interactions. Overall, the results presented here demonstrate the potential of targeting this universal viral entry pathway.

## 1. Introduction

Orthoflaviviruses such as Zika virus (ZIKV) pose serious threats to global human health. ZIKV is a positive-sense single-stranded RNA virus related to other pathogenic Orthoflaviviruses such as dengue virus (DENV), yellow fever, and West Nile virus. The 2015–2016 outbreak has demonstrated that ZIKV can cause severe epidemics. Since then, almost 1,000,000 autochthonous cases have been reported in the Americas [1]. Although infections mostly transmit asymptomatically or with only mild symptoms, ZIKV can cause serious neurological syndromes and lead to birth defects or even fetal loss [2,3,4,5]. Orthoflavivirus spread is changing and increasing due to several factors such as global warming and altered human land use, which elevates the spread of the mosquito vector *Aedes* spp. to more temperate regions [6,7,8]. Various potential antivirals are in clinical trials, but none of them are available on the market yet, making supportive care currently the main treatment [9,10,11]. This lack of treatment highlights the importance of the search for novel broad-spectrum antiviral targets.

This study focuses on the initial entry of ZIKV into the host cell, which is the first step of the viral replication cycle, described in detail elsewhere [12]. ZIKV enters target cells by interacting with membrane proteins, followed by clathrin-mediated endocytosis (CME) [13]. Different cellular factors have been found to be involved in Orthoflavivirus entry, one class being the phosphatidylserine (PS) receptors [14,15]. These normally bind to PS (and phosphatidylethanolamine [PE]) present on the outer cell membrane leaflet of apoptotic cells, thereby initiating phagocytosis [16]. Many viruses hijack this clearance machinery to invade cells, a process called apoptotic mimicry [17]. ZIKV, as well as many other enveloped viruses, exploits the PS receptor T-cell immunoglobulin mucin domain protein 1 (TIM-1) to enter host cells [18,19,20,21,22,23,24,25,26]. For example, it has been demonstrated that DENV co-internalizes with TIM-1 during CME [27]. TIM-1 plays an important costimulatory role in adequate immune responses [28,29,30]. The PS receptor CD300a plays a more inhibitory role in immune regulation [31,32]. This receptor was discovered to be involved in the entry of several Orthoflaviviruses, but its role in ZIKV infection remained unknown [33,34].

Although the virus-host interactions and subsequent pathophysiological mechanisms of ZIKV infection are not yet completely understood and can be strain specific, the virus is known to cause cell death in vitro [35,36,37]. Previous reports support the use of cell-based electrical impedance (CEI) to study Orthoflavivirus infection [38,39,40]. In this study, CEI is used to monitor the role of PS receptors as entry receptors for ZIKV infection. The cytopathogenic effects (CPE) caused by the virus are characterized by morphological changes, altered cell adherence, and eventually cell detachment, all leading to altered impedance measurements. Therefore, CEI is a straightforward method to monitor and quantify Orthoflavivirus infection. As PS receptors are involved in the entry of a broad range of viruses, they might be an attractive universal target in the search for a broad-spectrum antiviral drug. Here, we evaluate for the first time if CD300a is also involved in the in vitro ZIKV infection of HEK293T cells and monocyte-derived dendritic cells. TIM-1 plays an important role in the phagocytosis of apoptotic cells. Hence, we validate whether TIM-1 repression can affect ZIKV infection without changing the physiological ability of the cells to perform phagocytosis.

## 2. Materials and Methods

### 2.1. Cell Lines, Primary Cells, and Viruses

Cell lines. All used cells were obtained from American Type Culture Collection (ATCC, Manassas, VA, USA) unless stated otherwise. Human lung carcinoma A549 cells (cat. no. CCL-185) were cultured in Ham’s F-12K medium (Thermo Fisher Scientific [TFS], Waltham, MA, USA) supplemented with 5% fetal bovine serum (FBS; Cytiva, Marlborough, MA, USA). Baby hamster kidney BHK-21J fibroblast cells were kindly provided by P. Bredenbeek (LUMC, Leiden, The Netherlands) and cultured in Dulbecco’s modified Eagle’s medium (DMEM; TFS) supplemented with 8% FBS, 0.01 M HEPES (TFS) and 0.075% sodium pyruvate (TFS). C6/36 mosquito cells (cat. no. CRL-1660) were grown in Leibovitz’s L-15 medium containing 10% FBS, 0.01 M HEPES, and non-essential amino acids (TFS). Human embryonic kidney epithelium HEK293T cells (cat. no. CRL-3216) were maintained in DMEM supplemented with 8% FBS and 0.01 M HEPES. Chinese hamster ovary CHO.k1 cells (cat. no. CCL-61) were cultured in Ham’s F12 medium supplemented with 10% FBS. Cells were maintained at 37 °C in a humidified atmosphere supplemented with 5% CO_2_, except for C6/36 cells, which were kept at 28 °C in the absence of CO_2_. Every 3 to 4 days, cells were subcultivated.

Primary cells. Buffy coats from healthy donors were obtained from the Red Cross (Mechelen, Belgium, after receiving informed consent). Erythrocytes were removed using HetaSep (Stemcell Technologies, Vancouver, BC, Canada) and human peripheral blood mononuclear cells (PBMCs) were isolated using density gradient centrifugation over Lymphoprep (Stemcell Technologies). Subsequently, PBMCs were gently rotated at 4 °C to form monocyte aggregations. The obtained pellet was cultured in RPMI-1640 medium (TFS) supplemented with 10% FBS, 2 mM L-glutamine, 25 ng/mL IL-4, and 50 ng/mL GM-CSF (Peprotech, London, UK) to stimulate differentiation to immature monocyte-derived dendritic cells (MDDCs). Five days after stimulation, MDDCs were phenotypically characterized using flow cytometry [41] and infected.

Viruses. ZIKV MR766 prototype strain was obtained from ATCC (VR-84). Viral stocks were propagated in C6/36 cells and viral titers were determined by plaque assay in BHK-21J cells.

### 2.2. Molecular Cloning and Stable Cell Line Transfection

The shuttle vectors expressing human TIM-1 or CD300a cDNA inserts were obtained from R&D Systems (Minneapolis, MN, USA). The pBABE-Puro retroviral expression vector was purchased from Addgene (Watertown, MA, USA) [42]. Overlapping DNA ends in insert and expression vectors were introduced using PCR and insert cDNA was integrated into the expression vector using NEBuilder HiFi DNA Assembly Master Mix (New England Biolabs (NEB), Ipswich, MA, USA) according to the manufacturer’s guidelines. The assembled vector was transformed into NEB5α competent *E. coli* (NEB), and bacteria were grown on lysogeny broth (LB) agar plates containing 100 µg/mL ampicillin (Merck, Darmstadt, Germany). The next day, single colonies were selected and grown overnight in liquid LB medium with ampicillin. The Wizard Plus SV Miniprep DNA Purification System (Promega, Madison, WI, USA) was used to isolate plasmid DNA. To verify correct insert incorporation, plasmid DNA was sequenced by Macrogen (Amsterdam, The Netherlands).

To obtain TIM-1 or CD300a stably expressing HEK293T and CHO.k1 cells, cells were seeded and allowed to adhere overnight. They were transfected by adding TIM-1 or CD300a plasmid DNA and FuGENE HD (Promega, Madison, WI, USA) mixture in Opti-MEM medium (TFS) in a 1:3 ratio. After overnight incubation, puromycin selection (2 µg/mL; Merck) was used to obtain a stable cell line. Cell sorting was performed to increase receptor expression. To this end, cells were stained with mouse monoclonal antibody (mAb) conjugated to PE (either TIM-1 clone 1D12 (Biolegend, San Diego, CA, USA) or CD300a clone E59.126 (Beckman Coulter, Indianapolis, IN, USA)) and cell sorting was performed in the KU Leuven FACS Core with BD FACSMelody (BD Biosciences, Franklin Lakes, NJ, USA). The sorted populations were further cultured, and receptor expression was regularly verified using flow cytometry. Cells were collected and washed in Dulbecco’s phosphate buffered saline (DPBS) containing 2% FBS. They were incubated with either mouse anti-TIM-1 Ab (clone 1D12; Biolegend) or mouse anti-CD300a mAb (clone E59.126; Beckman Coulter, Brea, CA, USA) for 30 min at 4 °C in the dark. After washing, cells were fixed in 1% paraformaldehyde solution (Merck) and analyzed using BD FACSCelesta (BD Biosciences). Data were analyzed using FlowJo software Version 10.10 (BD Biosciences).

### 2.3. Genome Editing

A CRISPR/Cas9-mediated TIM-1 (also called Hepatitis A virus cellular receptor 1 [HAVCR]) knockout pool A549 cell line was generated using four different guides per gene selected from the Human Brunello CRISPR knockout pooled library (Table 1) [43]. As a negative control, a sgRNA targeting the AAVS1 safe harbor locus was used. Guide sequences were cloned into the pLentiCRISPRv2 plasmid (52961; Addgene) according to the standard cloning protocol. For lentiviral particle production, HEK293T cells (received from Prof. Jason Moffat, Donnelly Centre, University of Toronto, Toronto, ON, Canada) were plated in 40 mL supplemented DMEM in T150 (TPP, Trasadingen, Switzerland) flasks at 45% confluency and incubated overnight. Twenty-four hours later, the cells were co-transfected using X-TremeGENE 9 (Roche, Basel, Switzerland) with pLentiCRISPR plasmids and the lentiviral packaging plasmids pMD2.G and psPAX2 to generate lentiviral particles coated with the VSV-G protein. Twenty-four hours post-transfection, the medium was changed to DMEM supplemented with serum-free BSA growth media (DMEM + 1.1 g/100 mL BSA and 20 µg/mL gentamicin). The supernatant containing lentiviral particles was harvested 72 h after transfection and stored at −80 °C. Cells were transduced with lentiviruses expressing a pool of the 4 sgRNAs and then selected with puromycin for 3 days.

### 2.4. Validation of Knockout Cells

TIM-1 expression in gene edited A549 was verified using flow cytometry, Western blot, and quantitative PCR. Membrane receptor expression was quantified using flow cytometry as described above. Total TIM-1 protein was determined with immunoblotting based on a previous protocol [46]. Cells were lysed on ice in Nonidet P-40 buffer (NP-40; TFS, supplemented with 50 mM Tris HCl, pH 8.0, 150 mM NaCl, protease inhibitor cocktail (Roche) and phenylmethanesulfonyl fluoride (PMSF; Merck), final concentration 1%. Nuclei and debris were removed by centrifuging at 17,000× *g* for 10 min at 4 °C. After incubating samples for 10 min at 95 °C in reducing sample buffer (120 mM Tris-HCl, pH 6.8, 4% SDS, 20% glycerol, 100 mM dithiothreitol (Merck) and 0.02% bromophenol blue), they were run on a 4–12% Bis-Tris gel (Bio-Rad, Hercules, CA, USA) and blotted on a PVDF membrane (Bio-Rad) using the Trans-Blot Turbo system (Bio-Rad). Samples were blocked in 5% non-fat milk in TBST before incubating overnight at 4 °C with polyclonal goat anti-human TIM-1 antibody (R&D Systems) or clathrin (BD Biosciences). After thorough washing in TBST, blots were incubated for 1 h in HRP-conjugated donkey anti-goat antibody (TFS). After a final washing step, bands were detected using SuperSignal West Femto chemiluminescence reagent (TFS) and analyzed with a ChemiDocMP (Bio-Rad).

Quantitative RT-PCR was performed to quantify TIM-1 gene expression in knockout cells. RNA was extracted using an RNeasy kit (Qiagen, Hilden, Germany) and cDNA was made with the High-Capacity cDNA reverse transcription kit (TFS). RT-qPCR was performed using GoTaq qPCR master mix (Promega). The included A549 reference genes *B2M*, *18S* and *HPRT1* were based on the literature (Table 1) [47] and primer efficiencies were determined prior to gene expression determination. To calculate the relative TIM-1 gene expression, the ΔΔCt method was used.

### 2.5. Cell-Based Electrical Impedance Assay

The cell-based electrical impedance (CEI) assay was performed as described previously [48]. In short, cells were seeded in a pre-washed 96-well plate with interdigitated electrodes (Applied Biophysics, Troy, NY, USA) and allowed to adhere overnight in the ECIS Z device (Applied Biophysics) to measure impedance in multifrequency mode. After optional preincubation for 30 min with various concentrations of pAbs targeting either TIM-1 or CD300a (R&D Systems), cells were infected with ZIKV MR766 at different multiplicities of infection (MOI). Mock-infected untreated CC and untreated VC conditions were included. Impedance was monitored for six consecutive days. Measured impedance values (Z, Ohm, measured at 16,000 Hz) were extracted to Microsoft Excel to further analyze. Data were normalized by subtracting CC impedance values measured at the last time point before infection from each data point and dividing by VC impedance values measured at the end of the experiment. To quantify infection, area under the normalized curve (AUC_n_) was calculated with GraphPad Prism Version 10.1.1 (Graphpad Software Inc., San Diego, CA, USA), as well as maximal impedance values (Z_max_) and CIT_50_, defined as the time needed for normalized impedance to drop 50% compared to the uninfected cell control [36]. When the cell monolayer was altered to such an extent that impedance never reached this point, CIT_50_ was set at 0. When the cell monolayer remained stable and impedance did not decrease to reach CIT_50_ before the end of the experiment either, it was set to the maximum time of the experiment, i.e., 144 h.

### 2.6. Quantitative RT-PCR of Viral RNA

HEK293T, HEK293T TIM-1, HEK293T CD300a, CHO.k1, CHO.k1 TIM-1, CHO.k1 CD300a, A549 AAVS1 KO, or A549 TIM-1 KO cells were seeded at 15 × 10^5^ cells/well in cell culture medium in a 96-well plate. After overnight incubation, medium was replaced by assay medium (2% FBS) with or without anti-TIM-1/anti-CD300a pAb (10 µg/mL or a concentration range). Cells were subsequently infected with ZIKV MR766 at various MOI for 1 h. After washing, they were further incubated for 1 to 4 days in assay medium. Supernatant was collected, subjected to lysis (TFS), and RT-qPCR was performed using the CellsDirect One-Step qRT-PCR kit, following manufacturer’s instructions. Table 1 gives an overview of the used primers and probe.

### 2.7. Viral Plaque Assay

To determine viral titers after receptor transfection, HEK293T, HEK293T TIM-1, HEK293T CD300a, CHO.k1, or CHO.k1 CD300a cells were seeded in cell culture medium. After overnight incubation, cells were subsequently infected with ZIKV MR766 at various MOI diluted in assay medium (2% FBS). After 1 h incubation, unbound virus was removed, and cells were further incubated in assay medium for 24 h. Then, viral titer in the supernatant was determined using the plaque assay. To this end, BHK-21J cells were seeded in 12-well plates to reach a confluent monolayer and infected with triplicates of 10-fold dilutions of supernatant. After 1 h, cells were washed thoroughly and incubated for 4 days in a 1:1 mixture of assay medium and microcrystalline cellulose overlay (Avicel RC 581; IMCD Benelux, Mechelen, Belgium) [49]. After removing the overlay, cells were fixed in 70% ethanol and stained with Crystal Violet (Merck). Viral titer was calculated as the average number of plaques divided by dilution and inoculation volume.

### 2.8. MDDC Infection Assay

Immature dendritic cells (MDDC) were treated for 30 min with either polyclonal anti-CD300a antibody (10 µg/mL; R&D Systems), monoclonal anti-DC-SIGN antibody (10 µg/mL; TFS), a combination of both, or IgG isotype control. Subsequently, they were infected with ZIKV MR766 (MOI 0.1). One hour after infection, unbound virus was removed and cells were further cultured in assay medium for 48 h. Cells were collected, washed, and subjected to lysis (TFS) for subsequent viral RNA quantification through one step RT-qPCR (CellsDirect One-Step qRT-PCR kit, TFS). Percentage infection was normalized to isotype treated cells.

### 2.9. Phagocytosis Assay

This protocol was adapted from Miksa et al. (2009) [50]. Jurkat cells (1 × 10^6^ cells/mL; ATCC) were treated with Camptothecin (50 µM; Merck) for 24 h to induce apoptosis. A549 AAVS1 or A549 TIM-1 were seeded in 24-well plates until confluency was reached overnight. Apoptotic Jurkat cells were incubated with pHrodo Red succinimidyl ester (200 ng/mL and per 10^6^ Jurkat cells; TFS) for 30 min at RT. They were subsequently washed in serum-free medium and added to the A549 cells. Plates were incubated at 37 °C for 1, 2, or 3 h to allow phagocytosis of the Jurkat cells. Cells were detached, washed, and fixed in 1% PFA (Merck) before being analyzed by flow cytometry using a FACSCelesta (BD Biosciences). pHrodo red dye only emits fluorescent light when localized in the acidic environment of the phagolysosome, so the amount of phagocytosis was calculated by the fluorescence of A549 cells that engulfed apoptotic Jurkat cells. As a control, the fluorescence of A549 cells that remained at 4 °C during Jurkat addition was also measured.

### 2.10. Statistical Analysis

Statistical analyses were performed with GraphPad Prism. Variation between independent biological replicates was represented by standard deviation (SD) and variation between technical duplicates was depicted with range. To compare the means of two groups (i.e., untransfected vs. transfected or vehicle-treated vs. antibody-treated), two-tailed one sample *t*-test was used and *p*-values < 0.05 were indicated with asterisks.

## 3. Results and Discussion

### 3.1. TIM-1 and CD300a Transfection Increase ZIKV Infection in HEK293T Cells

**Figure 1:**
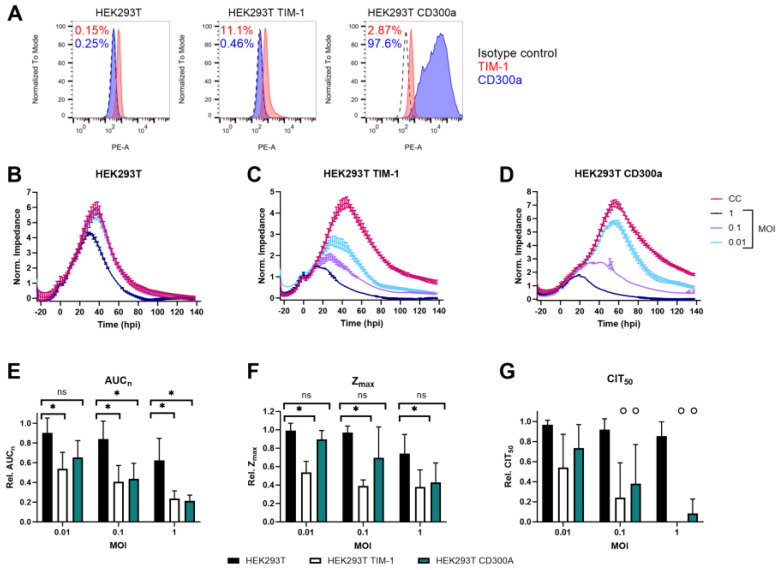
TIM-1 and CD300a expression increase ZIKV MR766 infection. (**A**) Cell surface expression levels of TIM-1 and CD300a of HEK293T cells that were untransfected (left) or transfected with TIM-1 (middle) or CD300a (right) plasmids, as determined by flow cytometry using TIM-1 (red) or CD300a (blue) antibodies. Percentages of cells expressing the respective receptor are indicated in red (TIM-1) or blue (CD300a). (**B**–**D**) CEI profiles of HEK293T, HEK293T TIM-1, and HEK293T CD300a cells infected with indicated MOI of ZIKV MR766. Impedance was monitored for several days after infection. The graphical representation of the normalized impedance pattern as a function of time is shown. The experiments were performed three to five times, and the results of one representative experiment with two technical duplicates are shown (mean ± range). Hpi: hours post infection. CC: uninfected cell control. (**E**–**G**) Calculation of various CEI parameters. Relative AUC_n_ (**E**), Z_max_ (**F**), and CIT_50_ (**G**) were calculated by comparing to the respective cell control values. Mean ± SD of three to five biological replicates, all with two technical duplicates, is shown. To compare untransfected with either TIM-1 or CD300a transfected HEK293T cells, two-tailed unpaired *t*-tests were performed. *p*-values < 0.05 are indicated with asterisks. ns, not significant. In (**G**), ‘o’ means that in one or more biological replicate(s), impedance of the condition dropped before CIT_50_ was reached. In this case, CIT_50_ was set to 0. Therefore, no statistics were performed for these conditions.

HEK293T cells were chosen for the PS expression studies since they are ideally suited for transfection and relatively resistant to Orthoflavivirus infection [18]. Their cancerous nature makes them easier to accept foreign macromolecules such as DNA. Furthermore, their ability to adhere to surfaces increases cell-to-cell contact, which makes HEK293T cells a popular choice for protein expression and recombinant protein production. They were stably transfected with either TIM-1 or CD300a (Figure 1A) and infected with ZIKV MR766 at different multiplicities of infection (MOI). The consequences of infection on the cell monolayer were evaluated using real-time CEI measurements. Considering that transfected HEK293T expressed different levels of TIM-1 and CD300a (Figure 1A), we could not make any direct comparisons between the receptors to determine their relative role in ZIKV entry.

However, ZIKV infection between wild-type and transfected cells can be compared. As shown in Figure 1B, wild-type cells were rather resistant to infection, as the monolayer was only affected by ZIKV at the highest tested MOI (i.e., one). Of note, all HEK293T cells were infected before confluent monolayers were reached, since these cells tend to overgrow quickly, leading to impedance being more affected due to biological reasons rather than due to viral infection. This explains the initial steep increase in impedance after infection, as cells were still in their exponential growth phase. Furthermore, the intercellular space of HEK293T does not contain any tight junctions, which can influence cellular impedance [51]. It would be interesting to compare the impedance profiles of HEK293T after ZIKV infection at different frequencies to confirm if the observed effects are indeed caused by viral entry

When TIM-1 expression was slightly increased, infection also increased and the cell monolayer was affected in a MOI-dependent way, as reflected in lower and shorter CEI responses (Figure 1C). This was expected based on previous reports [18]. When CD300a was expressed, ZIKV infection increased as well (Figure 1D). This is the first time the involvement of CD300a in ZIKV infection has been demonstrated.

To assess ZIKV infection kinetics more quantitatively, several CEI parameters were calculated, as indicated in Figure S1. First, the AUC_n_ was calculated as the integral of all normalized impedance values at each timepoint [52]. The AUC_n_ is a measure of the growth and viability of the cells with or without ZIKV exposure (Figure 1E). In general, the higher the MOI, the more affected the cells, as illustrated by the decreasing AUC_n_. TIM-1 or CD300a expression clearly enhanced ZIKV infection compared to wild-type cells. Only at the lowest tested MOI (0.01) was the difference not significant in HEK293T CD300a cells.

Second, the maximal impedance values (Z_max_) were compared (Figure 1F). This is a measure of the maximal reached cell confluency in infected or uninfected cells, and it is in accordance with microscopic observations. TIM-1 expression significantly decreased the relative Z_max_ of ZIKV-infected cells, indicating that the cell monolayer was destructed by the virus before maximal confluency could be reached. In addition, when CD300a was expressed, Z_max_ decreased after ZIKV infection, albeit in a non-significant way. This indicates that the cells had more time to form a monolayer before the cytopathogenic effects of viral replication began.

Third, CIT_50_ values were calculated and compared. This parameter is defined as the time point at which normalized impedance has dropped by 50% compared to the uninfected cell control. It is a measure of how fast infection affects the cells by inducing morphological changes or detachment [39]. However, as ZIKV infection caused fast disruption of the cell monolayer in HEK293T TIM-1 and HEK293T CD300a cells, impedance dropped so fast that no 50% of CC Z_max_ was reached, and no CIT_50_ could be calculated. To be able to visualize this, CIT_50_ was set to 0 in this case (Figure 1G). This effect was observed at every MOI, except when HEK293T CD300a cells were infected at the lowest tested MOI of 0.01. Of course, no statistics are possible to demonstrate the differences between transfected and untransfected cells, but the fast impedance drop demonstrates that TIM-1 or CD300a expressing cells are more sensitive to ZIKV infection. Importantly, this observation does point out that CIT_50_ is not an ideal parameter in all CEI studies.

Altogether, these results indicate that ZIKV MR766 entry is enhanced when TIM-1 or CD300a are expressed. Cytopathogenic effects are more pronounced and commence earlier than in wild-type cells. This can all be monitored in real time and quantified using CEI.

**Figure 2:**
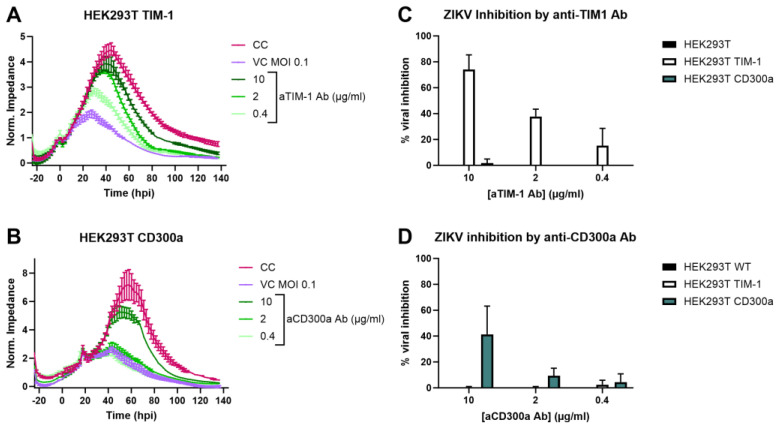
ZIKV infection increase in HEK293T cells is due to TIM-1 or CD300a expression. (**A**,**B**) Seeded HEK293T TIM-1 (**A**) or HEK293T CD300a (**B**) cells were treated with various concentrations of anti-TIM-1 Ab or anti-CD300a Ab, respectively (R&D Systems), and subsequently infected with ZIKV MR766 MOI 0.1. Infection was monitored using CEI, and normalized impedance profiles were plotted over time. The experiment was performed three times, and the results of one representative experiment performed in duplicate are shown (mean ± range). CC: vehicle-treated, mock-infected cell control. VC: vehicle-treated virus control. (**C**,**D**) AUC_n_ of cells treated with either anti-TIM-1 Ab (**C**) or anti-CD300a Ab (**D**) was calculated and compared to AUC_n_ of CC (set to 100%) and VC (set to 0%) to calculate the percentage viral inhibition. Mean ± SD of three biological replicates each performed with two technical duplicates is shown.

To confirm that this infection increase was due to the specific expression of TIM-1 or CD300a, cells were pre-incubated with antibodies targeting the respective receptors and subsequently infected with ZIKV MR766 MOI 0.1. The infection was monitored using CEI. As shown in Figure 2A,B, infection decreased in a dose-dependent way after Ab treatment. This effect was specific, as infection was unaltered when wild-type or CD300a transfected cells were treated with anti-TIM-1 Ab, or vice versa for the anti-CD300a Ab (Figure 2C,D and Figure S2).

**Figure 3:**
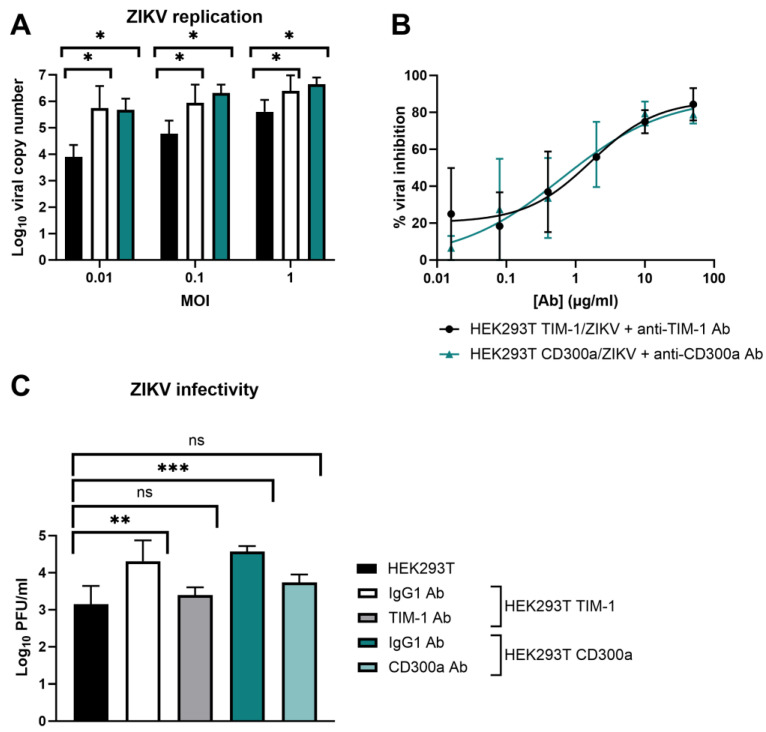
ZIKV replication and infectivity is enhanced when TIM-1 or CD300a is expressed. (**A**) HEK293T, HEK293T TIM-1, and HEK293T CD300a cells were allowed to adhere overnight and infected with ZIKV MR766 at various MOI. After one hour, cells were washed and further incubated for three days. Viral genome copies in the supernatant were determined using RT-qPCR. (**B**) Same as A, but cells were infected with ZIKV MOI 0.1 in the presence or absence of various concentrations of anti-TIM-1 Ab or anti-CD300a Ab (R&D Systems). The percentage inhibition of an Ab concentration was obtained by normalizing to the copy number in vehicle-treated VC. Dose-response curves were obtained using the nonlinear regression fit tool in GraphPad Prism. (**C**) HEK293T, HEK293T TIM-1, and HEK293T CD300a cells were allowed to adhere overnight and treated with anti-IgG, anti-TIM-1, or anti-CD300a Ab (10 µg/mL) prior to infection with ZIKV MR766 (MOI 1) for one hour. After thorough washing, cells were further incubated for 24 h, and viral titers in the supernatant were determined with plaque assay in BHK-21J cells. (**A**–**C**) Mean ± SD of three to five independent experiments performed in duplicate is shown. To compare conditions, two-tailed unpaired *t*-tests were performed. *p*-values < 0.05 are indicated with asterisks. ns, not significant.

Next, we determined if, aside from enhanced entry, ZIKV replication and production of the infectious virus is also altered after TIM-1 and CD300a transfection. Therefore, the presence of ZIKV genomes in the transfected or untransfected cell supernatant was determined by RT-qPCR, and the infectivity of these newly formed virions was quantified by titration with a plaque assay (Figure 3). The results confirm the observations of the CEI experiments, i.e., ZIKV replication was significantly increased after TIM-1 or CD300a expression (Figure 3A). Pretreatment of the cells with anti-TIM-1 or anti-CD300a Abs inhibited viral replication in a dose-dependent way, again confirming that the ZIKV replication increase was due to PS receptor expression (Figure 3B). The infectivity of the viral progeny also increased after TIM-1 and CD300a transfection, and this could also be prohibited when cells were pretreated with receptor-specific Abs (Figure 3C). In summary, ZIKV entry, replication, and infectivity is enhanced when the PS receptors TIM-1 or CD300a are expressed. This effect is independent of MOI. The results obtained using the traditional methods of RT-qPCR and viral titration validate the use of CEI as an appealing tool in entry receptor studies.

**Figure 4:**
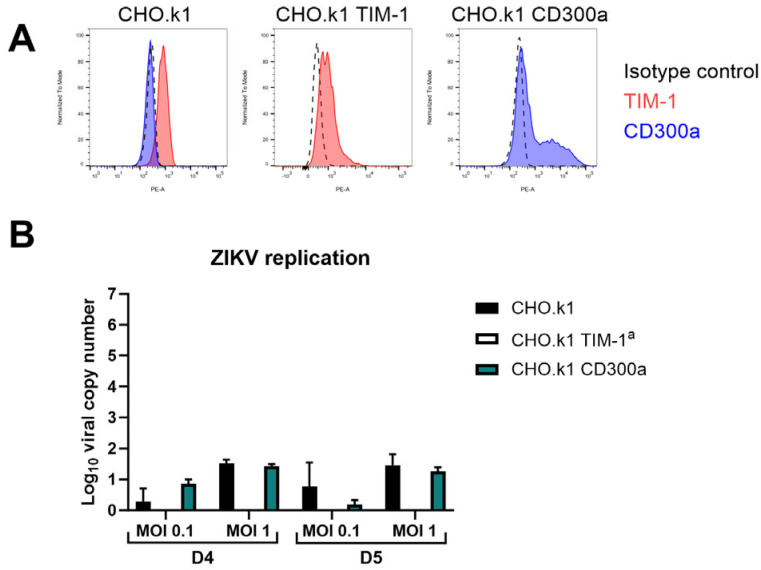
CHO.k1 cells remain unpermissive to ZIKV infection after TIM-1 or CD300a transfection. (**A**) Cell surface expression levels of TIM-1 and CD300a of CHO.k1 cells that were untransfected (left) or transfected with TIM-1 (middle) or CD300a (right) plasmids, as determined by flow cytometry using TIM-1 (red) or CD300a (blue) antibodies. (**B**) CHO.k1, CHO.k1 TIM-1, or CHO.k1 CD300a cells were infected with ZIKV MR766 at MOI 0.1 or MOI 1 for one hour. After thorough washing, they were further incubated for four (D4) or five (D5) days and viral copy number in the supernatant was determined using RT-qPCR. Mean ± SD of one experiment performed in duplicate is shown. ^a^ No viral copy number could be calculated since results were below the limit of detection. In the viral plaque assay, seeded CHO.k1 or CHO.k1 CD300a cells were infected with ZIKVMR766 M0I 1 for one hour. After thorough washing, they were further incubated for 24 h and viral titer in the supernatant was determined on BHK-21J cells using plaque assay. No plaques were detected in either of the two biological replicates, so these results are not shown.

It is important to note that solely expression of the PS receptors does not suffice to make cells susceptible to ZIKV infection. As shown in Figure 4, cell surface expression of TIM-1 or CD300a on CHO.k1 cells, a hamster cell line known to be unsusceptible to ZIKV infection, does not increase replication of the virus [53]. The viral titer in the supernatant did not increase compared to untransfected cells (no plaques were observed), and no viral genomes were released. This suggests that other entry factors or alternative post-entry steps are necessary as well.

### 3.2. TIM-1 Knockout Decreases ZIKV Entry

**Figure 5:**
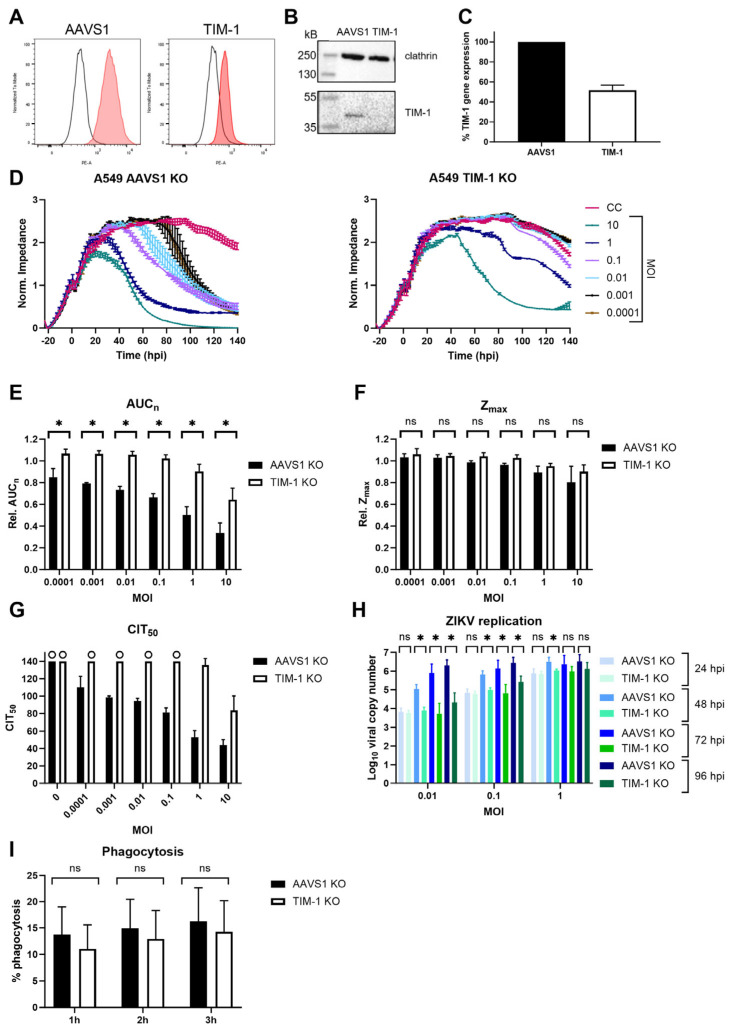
TIM-1 expression knockout in A549 cells decreases ZIKV MR766 infection. (**A**) KO pool cell line of AAVS1 safe harbor locus (‘AAVS1 KO’) or TIM-1 (‘TIM-1 KO’) was engineered and validated by determining TIM-1 expression using flow cytometry (**A**), Western blot (**B**), and RT-qPCR (**C**). (**C**) Percentage TIM-1 gene expression was calculated according to the ΔΔCt method including three reference genes and normalized to AAVS1 KO negative control A549 cells. Mean ± SD of three independent replicates (each performed in triplicate) is shown. (**D**) AAVS1 (left panel) or TIM-1 (right panel) KO A549 cells were infected with various MOI of ZIKV and the CEI profile of the cells was monitored. CEI profiles of a representative experiment (mean ± range of 2 technical replicates) are shown. Hpi: hours post infection. CC: mock-infected cell control. (**E**,**F**) The area under the normalized curve (AUC_n_) and maximal impedance (Z_max_) of these experiments (three biological replicates each with two technical duplicates) were calculated and compared relative to CC (rel. AUC_n_, rel. Z_max_). (**G**) CIT_50_ of these experiments (three biological replicates each with two technical duplicates) was also calculated. When no CIT_50_ could be calculated, since impedance was still too high, the end of the experiment, i.e., 140 hpi, was used. This was the case when ‘o’ is shown in the graph. (**H**) AAVS1 or TIM-1 KO pool A549 cells were allowed to adhere overnight and infected with various MOI of ZIKV MR766. One to four days after infection, viral copy number in the supernatant was determined using RT-qPCR. Mean ± SD of three experiments performed in duplicate is shown. (**I**) A549 AAVS1 KO or A549 TIM-1 KO were grown to confluency and incubated with pHRodo red-stained apoptotic Jurkat cells for one, two, or three hours at 37 °C. Percentage phagocytosis was measured by counting the fluorescent cells with flow cytometry. Mean ± SD of four independent experiments performed in triplicate is shown. In (**E**,**F**,**H**,**I**), AAVS1 and TIM-1 KO cells were compared using two-tailed unpaired *t*-tests. *p*-values < 0.05 are indicated with asterisks. ns, not significant. No statistics were performed in (**G**), because for several conditions, no CIT_50_ could be calculated.

To investigate ZIKV infection when TIM-1 expression levels are decreased, a CRISPR-Cas9-mediated polyclonal knockout (KO) pool A549 cell line was engineered (‘A549 TIM-1’). As a control, A549 AAVS1 KO cells were also generated. This A549 cell line was chosen as it is commonly used in Orthoflavivirus research and endogenously expresses TIM-1 [20]. Figure 5A,B show that TIM-1 protein expression decreased after editing, and gene expression levels decreased by 50% (Figure 5C). The CEI profiles of mock-infected control cells show that A549 cells have a different growth pattern than HEK293T cells. After having reached confluency, they remain in a stable monolayer for several days. When A549 AAVS1 KO cells were infected with ZIKV MR766, the consequences of infection on the cell monolayer were monitored with CEI until MOI 0.0001. Although TIM-1 expression was only partly decreased in A549 TIM-1 KO cells, a remarkable CEI profile change was observed compared to A549 AAVS1 KO negative control cells after ZIKV infection (Figure 5D). At the highest MOI of 10 and 1, the cell monolayer was still destructed, albeit at a slower rate. At lower MOIs, the CEI profile of A549 TIM-1 KO cells followed the same pattern as mock-infected control cells.

The effect of TIM-1 expression decrease was also confirmed by calculating the relative AUC_n_ (Figure 5E). When comparing relative Z_max_, no significant differences between A549 AAVS1 KO and TIM-1 KO were observed, indicating that monolayer confluency was reached before infection caused impedance changes (Figure 5F). Only at the highest MOI of 10 and 1 was Z_max_ lower in infected than in mock-infected cells, but this was comparable to AAVS1 and TIM-1 KO pool cells. Calculating CIT_50_ values in AAVS1 KO A549 cells (Figure 5G, black bars) indicated that the higher the MOI, the faster impedance decreased, as the cell monolayer was altered sooner. Knocking out TIM-1 slowed down ZIKV replication, as no difference was observed between mock-infected and infected cells (Figure 5G, white bars). Note that in mock-infected AAVS1 KO, TIM-1 KO A549, and in most infected A549 TIM-1 KO conditions, no CIT_50_ could be calculated (indicated with the maximum time of 140 hpi and ‘o’). This again indicates the suboptimal use of the CIT_50_ parameter in this kind of study.

Based on our experimental settings (i.e., epithelial cell lines with transfected receptors and a cytopathic virus) and results, we propose that overall, the best CEI parameter to quantify the role of entry receptors in ZIKV infection is the AUC_n_. This parameter integrates the continuous impedance measurements and the effect of viral infection on the cell monolayer. Z_max_ is highly dependent on the growing kinetics of the cells, as seen in the short Z_max_ peaks for HEK293T cells (Figure 1B), while more stable Z_max_ periods are observed for A549 cells (Figure 5D). Although the CIT_50_ is a very useful parameter because it is correlated to the viral titer [38], in CEI studies comparing the impact of certain receptors on viral infection with CPE-causing viruses, it is less interesting. Cells tend to suffer from pathogenic effects—indicated with impedance drops—before they reach the half-maximal impedance values of uninfected control cells. On the other hand, impedance of cells that form a monolayer for an extended time never decrease sufficiently to calculate the CIT_50_ parameter.

To evaluate if viral replication was also affected after TIM-1 KO, the viral copy number in the supernatant was determined at various days post-infection (Figure 5H). As expected, ZIKV replication increased with increasing MOI and infection time. One day after infection, ZIKV replication in TIM-1 KO pool cells was not significantly different from the negative control cells. However, two days after infection, ZIKV replication in TIM-1 KO cells was significantly lower than in AAVS1 KO cells. The same observation was made three and four days after infection, except for the highest tested MOI (i.e., one). This result could also be attributed to the fact that the maximal level of replication had been reached in AAVS1 KO cells at this MOI. Overall, integrating the obtained CEI and RT-qPCR results suggests that initial viral input leads to comparable virus levels after one replication round, but newly formed virions are less capable of infecting and replicating in new cells when these express less TIM-1.

To determine if a partial expression decrease also influences TIM-1 functioning, a phagocytosis assay was performed. TIM-1 plays an important role in the phagocytosis of apoptotic cells, and previous research has shown that expression of the receptor in epithelial cells increases uptake by apoptotic cells [33,54]. To this end, the uptake of apoptotic Jurkat cells was compared between A549 AAVS1 KO and A549 TIM-1 KO cells using a flow cytometry assay. The results show that phagocytosis in cells with lower TIM-1 expression was somewhat decreased, but not in a significant way (Figure 5I). The results presented here indicate that a partial TIM-1 KO decreased ZIKV infection without altering its function. Our results further support previous reports that targeting this receptor might be an appealing strategy to reduce ZIKV entry. To confirm these results in a more physiologically relevant model, similar experiments could be performed in the future using primary microvascular endothelial cell lines, for which CEI was previously used [55,56].

### 3.3. Inhibition of CD300a in MDDC Partially but Significantly Decreases ZIKV Replication

**Figure 6:**
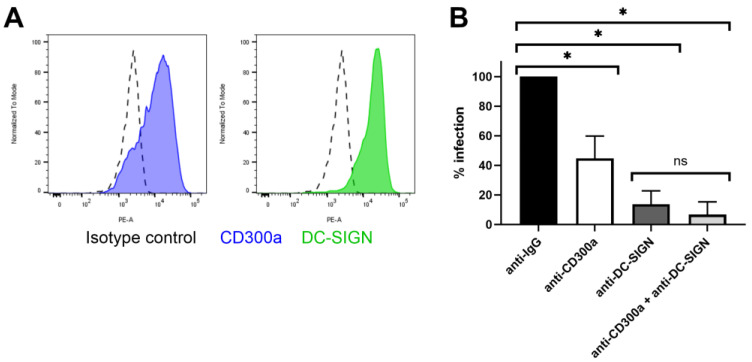
Treatment with anti-CD300a partially decreases ZIKV replication in MDDC. (**A**) MDDC derived from stimulated monocytes with IL-4 and GM-CSF were stained with either isotype control conjugated to PE, anti-CD300a-PE mAb or anti-DC-SIGN-PE mAb and analyzed with flow cytometry for detection of cell surface expression of the specific receptors. (**B**) MDDC were treated with anti IgG isotype control Ab, anti-CD300a pAb, anti-DC-SIGN mAb or a combination of both, at 10 µg/mL for 30 min. Cells were infected with ZIKV MOI 0.1 for one hour. After washing, cells were further incubated for 48 h prior to determining viral copy number in the cells using RT-qPCR. Percentage infection is normalized to the isotype treated condition. Mean ± SD of four independent buffy coat donors (each in duplicate) is shown. Data were compared using two-tailed unpaired *t*-tests. *p*-values < 0.05 are indicated with asterisks. ns, not significant.

The role of CD300a in DENV infection in primary cells has been well studied [33]. As this virus is closely related to ZIKV, we further investigated if this receptor is also important in ZIKV entry of primary dendritic cells. To this end, we isolated monocytes from the buffy coats of healthy donors and stimulated them with cytokines to obtain immature monocyte-derived dendritic cells (MDDC), as these cells are a physiologically relevant and validated model for ZIKV infection [57,58]. They are antigen-presenting cells serving as a primary target when the virus is transmitted after a mosquito bite. Furthermore, MDDC are known to express the CD300a receptor [31] (Figure 6A). When MDDC were treated with anti-CD300a Ab (10 µg/mL) prior to infection, ZIKV replication decreased partially but significantly (Figure 6B). This suggests that the virus can still enter the cells through interaction with another cellular factor. Another receptor expressed by immature MDDC and involved in ZIKV entry is Dendritic Cell-Specific Intercellular adhesion molecule-3-Grabbing Non-integrin (DC-SIGN) [18,59]. When pretreating MDDC with an antibody targeting this receptor, viral replication was almost completely inhibited. A combination treatment with both antibodies further decreased replication, albeit in an unsignificant way. Our results suggest that in MDDC, DC-SIGN is a more crucial cellular factor for ZIKV than CD300a. This is also supported by the recent study of Eder et al. (2023). Here, the authors suggest that dendritic cells in the skin and vaginal tract probably are an important primary target cell type for ZIKV due to the interaction between the virus and DC-SIGN [60]. This C-type lectin receptor is expressed on dendritic cells and macrophages, also enhancing the infection of other viruses such as DENV by interacting with the glycosylations present on their envelope. The receptor plays an important role in viral transmission and dissemination to other cell types when the dendritic cells travel to the lymph nodes to activate an antiviral immune response.

Overall, these results suggest that solely inhibiting CD300a does not suffice to protect dendritic cells from infection, but treatment with a compound targeting the receptors could aid in decreasing the virus concentration on the dendritic cell membrane, decreasing the interaction with DC-SIGN and further dissemination to other cell types.

Due to the clinical relevance of MDDC for studying ZIKV infection, an attractive future approach would be the incorporation of CEI in these experiments. However, working with MDDC using CEI can be problematic, as these cells are not completely adherent and not proliferating. Experimental conditions must be carefully optimized to provide reliable impedance readouts, including proper coating and high cell densities. In addition, inter-donor variation increases the need for more replicates, rendering these kinds of experimental studies less cost effective.

## 4. Conclusions

In this study, we used CEI biosensor technology to study the role of the PS receptors TIM-1 and CD300a in in vitro ZIKV infection using overexpression and knockout cell culture models. We show that CEI is a valuable tool to quantify the influence and kinetics of the increase or decrease of receptor expression on ZIKV infection. We also report for the first time that CD300a enhanced ZIKV infection, as has been previously shown for other Orthoflaviviruses as well. Blocking the receptor with an anti-CD300a antibody decreased ZIKV replication in a partial but significant way in primary dendritic cells. Finally, we demonstrated that small changes in TIM-1 expression (decrease or increase) significantly increased and accelerated ZIKV entry and replication. Considering these observations, it would be of interest to evaluate the effects of other ZIKV strains and closely related Orthoflaviviruses, such as DENV and yellow fever virus, using a similar CEI-based approach. Overall, the presented data support the role of PS receptors TIM-1 and CD300a in ZIKV infection and provide insights into the possibility of developing PS inhibitors targeting this universal entry pathway [61,62].

## Figures and Tables

**Table 1:** Primers and probes used in this study.

Name ^1^	Sequence (5′ to 3′)	Reference
CRISPR-Cas9 A549		
AAVS1 sgRNA	GTCACCAATCCTGTCCCTAG	[43]
HAVCR1 sgRNA 1	GCTCGTTCGAACAGTCGTGA	
HAVCR1 sgRNA 2	CACACGCTATAAGCTATTGG	
HAVCR1 sgRNA 3	ATGTGACAGCTCCACTGTAG	
HAVCR1 sgRNA 4	ACCACCCACGGTGCTCAACA	
RT-qPCR gene expression		
TIM-1 1	CTTCACCTCAGCCACAGAAAC	na
TIM-1 2	GCCATCTGAGACTCTGTCACG	na
18S 1	GTTCCAGCATATTTTGCGAGT	na
18S 2	GTCAATGTCTGCTTTCCTCAAC	na
HPRT1 1	TTGTTGTAGGATATGCCCTTGA	na
HPRT1 2	GCGATGTCAATAGGACTCCAG	na
B2M 1	GGACTGGTCTTTTATCTCTTGT	na
B2M 2	ACCTCCATGATGCTGCTTAC	na
ZIKV RNA quantification		
ZIKV Forward	CCGCTGCCCAACACAAG	[44]	
ZIKV Reverse	CCACTAACGTTCTTTTGCAGACAT		
ZIKV Probe	FAM/AGCCTACCT/ZEN/TGACAAGCAATCA GACACTCAA/IBFQ	[45]	

^1^ All primers and probes were obtained from Integrated DNA Technologies (Coralville, IA, USA). na: not applicable.

## Data Availability

The data presented in this study are available on request from the corresponding authors.

## Supplementary Materials

The following supporting information can be downloaded at https://www.mdpi.com/article/10.3390/bios14080362/s1, Figure S1: Graphical representation of calculated CEI parameters; Figure S2: ZIKV infection increase after TIM-1 or CD300a expression is receptor-specific; Figure S3: Full Western blot image of A549 AAVS1 KO and A549 TIM-1 KO.
