# Supplementary material for: The Role of TIM-1 and CD300a in Zika Virus Infection Investigated with Cell-Based Electrical Impedance
Source: Biosensors (Basel). 2024 Jul 25;14(8):362. doi: 10.3390/bios14080362 (PMC11352571; doi:10.3390/bios14080362)
Supplement: Supplementary file 1 [file biosensors-14-00362-s001.zip › biosensors-3049174-supplementary.pdf]

Supplementary Materials

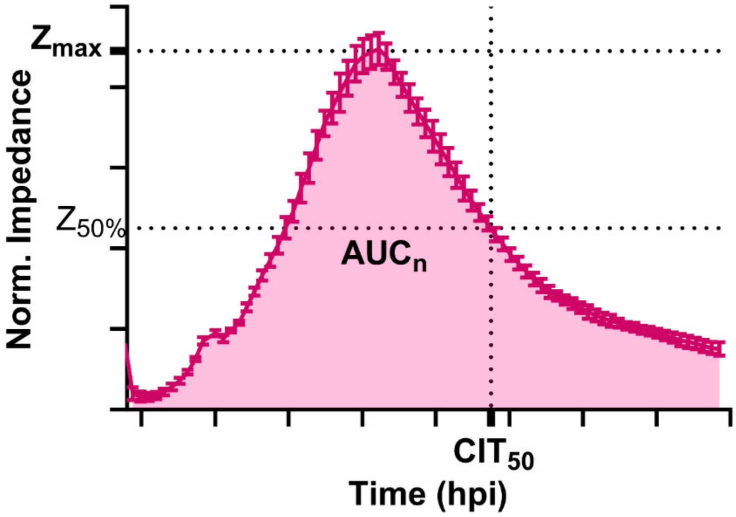

Supplementary Figure S1. Graphical representation of calculated CEI parameters. Hypothetical CEI profile of HEK293T cells with marks to visualize the determination of used CEI parameters. AUC_n: the normalized area under the impedance profile curve. Z_{max}: the maximal normalized impedance value. CIT_{50}: the time needed for impedance to drop with 50% compared to the maximum normalized impedance value (Z_{50}). Relative values (Rel. AUC_n, Rel. Z_{max} and Rel. CIT_{50}) were calculated by comparing the specific parameter to that of CC.

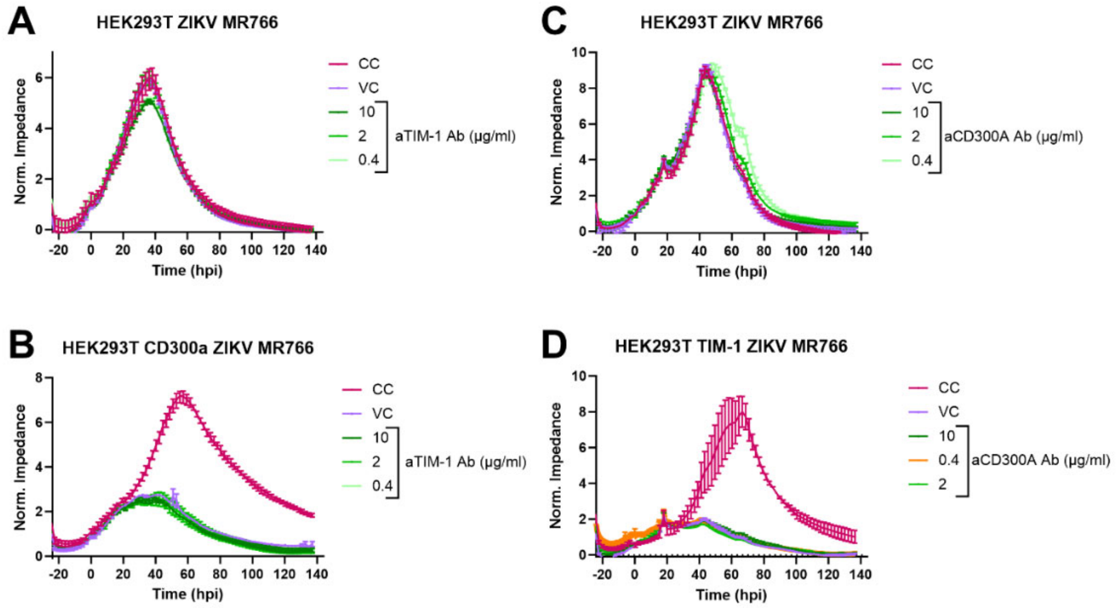

Supplementary Figure S2. ZIKV infection increase after TIM-1 or CD300a expression is receptor-specific. Seeded HEK293T (**A**) or HEK293T CD300a (**B**) cells were treated with various concentrations of anti-TIM-1 Ab and HEK293T (**C**) or HEK293T TIM-1 (**D**) were treated with various concentrations of anti-CD300a Ab. Cells were subsequently infected with ZIKV MR766 MOI 0.1 and infection was monitored using CEI. Normalized impedance profiles were plotted over time. Results of a representative experiment with two technical duplicates (mean ± range) are shown.

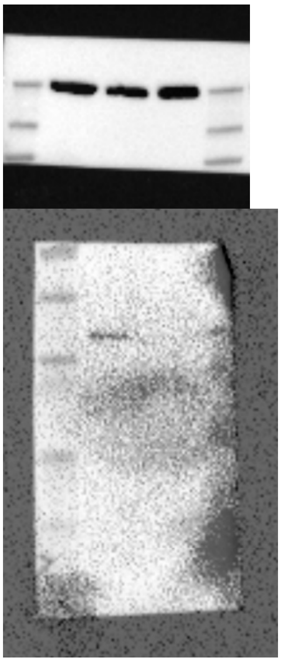

Supplementary Figure S3. Full Western blot image of A549 AAVS1 KO and A549 TIM-1 KO. KO pool cell line of AAVS1 safe harbor locus (first lane) or TIM-1 (second lane) was engineered and validated by determining TIM-1 expression using Western blot. The third lane contains a sample not used in this study. Top blot: clathrin expression, exposure time 14 s. Bottom blot: TIM-1 expression, exposure time 95 s.
